# In the Crosshairs: RNA Viruses OR Complement?

**DOI:** 10.3389/fimmu.2020.573583

**Published:** 2020-09-29

**Authors:** Nisha Asok Kumar, Umerali Kunnakkadan, Sabu Thomas, John Bernet Johnson

**Affiliations:** ^1^Viral Disease Biology, Department of Pathogen Biology, Rajiv Gandhi Center for Biotechnology, Thiruvananthapuram, India; ^2^Manipal Academy of Higher Education, Manipal, India; ^3^Department of Biotechnology, University of Kerala, Thiruvananthapuram, India; ^4^Cholera and Biofilm Research Lab, Department of Pathogen Biology, Rajiv Gandhi Center for Biotechnology, Thiruvananthapuram, India

**Keywords:** complement activation, viral evasion strategies, RNA viruses, complement regulators, virus neutralization

## Abstract

Complement, a part of the innate arm of the immune system, is integral to the frontline defense of the host against innumerable pathogens, which includes RNA viruses. Among the major groups of viruses, RNA viruses contribute significantly to the global mortality and morbidity index associated with viral infection. Despite multiple routes of entry adopted by these viruses, facing complement is inevitable. The initial interaction with complement and the nature of this interaction play an important role in determining host resistance versus susceptibility to the viral infection. Many RNA viruses are potent activators of complement, often resulting in virus neutralization. Yet, another facet of virus-induced activation is the exacerbation in pathogenesis contributing to the overall morbidity. The severity in disease and death associated with RNA virus infections shows a tip in the scale favoring viruses. Growing evidence suggest that like their DNA counterparts, RNA viruses have co-evolved to master ingenious strategies to remarkably restrict complement. Modulation of host genes involved in antiviral responses contributed prominently to the adoption of unique strategies to keep complement at bay, which included either down regulation of activation components (C3, C4) or up regulation of complement regulatory proteins. All this hints at a possible “hijacking” of the cross-talk mechanism of the host immune system. Enveloped RNA viruses have a selective advantage of not only modulating the host responses but also recruiting membrane-associated regulators of complement activation (RCAs). This review aims to highlight the significant progress in the understanding of RNA virus–complement interactions.

## Introduction

**FIGURE 1:**
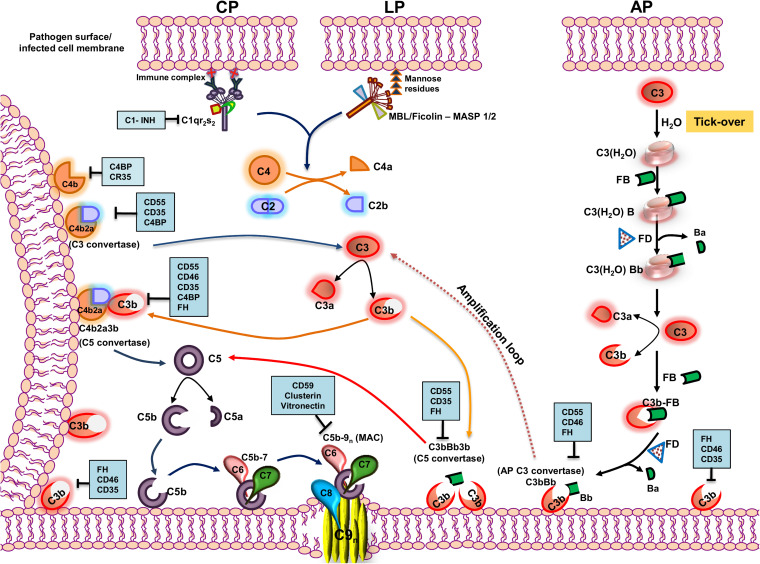
Pathways of complement activation with complement regulators and inhibitors highlighted. The complement system is activated primarily through three pathways—classical pathway (CP), lectin pathway (LP), and alternative pathway (AP). The CP is activated by the recognition of antigen–antibody complexes formed on the surface of the pathogen or pathogen-infected cells by the C1 complex (C1qr_2_s_2_), whereas the LP is activated by the recognition of specific carbohydrate moieties present on the surface of pathogens (PAMPs) by the MBL/ficolin–MASP 1/2 complex. Upon recognition, the complexes so formed cleave C4 and C2 into C4a and C4b and C2a and C2b, respectively. The C4b so formed binds to the membrane and associates with C2a to form the C4b2a complex, the CP/LP-C3 convertase. The C3 convertase cleaves C3 into C3a and C3b. The C3b so formed further associates with C4b2a to form the C5 convertase, C4b2a3b. AP, on the other hand, is constitutively active in the plasma by a mechanism called the “tick-over,” wherein the autohydrolysis of labile thioester bond in C3 forms C3(H_2_O). The latter binds to factor B and serine protease factor D. Factor D cleaves factor B into Ba and Bb and forms C3(H_2_O)Bb, the initial fluid-phase AP C3 convertase. It then cleaves C3 into C3a and C3b. The C3b associates further to factor B, and factor D cleaves factor B into Ba and Bb and results in the formation of surface-bound AP C3 convertase, C3bBb, stabilized by properdin. AP C3 convertase cleaves many native C3 molecules into C3a and C3b to form a positive amplification loop, with each C3b capable of forming new AP convertases. The newly formed C3b can also bind to C3bBb and forms C3bBb3b, which is the AP C5 convertase. All the pathways converge at the terminal step where the C5 convertase cleaves C5 into C5a and C5b. The C5b binds to the cell surface to which binds C6, C7, C8, and multiple molecules of C9, inserting itself into the membrane to form a multiprotein transmembrane pore called the membrane attack complex (MAC), which ultimately results in the lysis of the cell. The events in the activation cascade are regulated at various levels by a group of host proteins called the regulators of complement activation (RCAs). The regulators either can be soluble in nature (C1INH, C4BP, and FH) or are membrane-bound (CD55, CD46, CD35, and CD59). The regulatory proteins are highlighted within blue boxes. Key: CD55 (Decay accelerating factor, DAF), CD46 (Membrane cofactor protein, MCP), C1INH (C1 inhibitor), CD35 (Complement receptor 1, CR1), and FH (factor H).

The complement system (CS) is evolutionarily ancient and is a prime component of the innate immunity arsenal, capable of targeting a wide range of pathogens (1). Comprising of over 30 soluble and membrane-bound proteins, multiple functions besides anti-pathogenic activities have been attributed to the CS. Besides hepatocytes being the site of bulk synthesis of complement proteins in circulation, many other cell types including immune cells (2), adipocytes, fibroblasts, and endothelial cells also synthesize complement proteins locally as reviewed in Morgan and Gasque (3) imparting their autocrine and paracrine functions (4). The CS operates via three pathways, namely, the classical pathway (CP), the lectin pathway (LP), and the alternative pathway (AP) (5), and functions like a cascade brought about by the sequential proteolytic cleavage of zymogens into their active fragments. Despite differences in the initial trigger, all three pathways converge at a common factor, C3, quintessential for the complement cascade to proceed. The activation of a specific pathway depends on the recognition of unique molecules associated with the pathogen. The CP is initiated by the binding of C1q to the Fc region of IgG or IgM in an antigen–antibody complex or directly to the pathogen. This leads to the autocatalytic activation of C1r, which activates the serine protease C1s, which can then sequentially cleave C4 into C4a and C4b and C2 into C2a and C2b (6). In contrast, binding of mannose binding lectin (MBL) or ficolins to unique carbohydrate moieties rich in D-mannose or L-fucose (7) on the pathogen surface [pathogen-associated molecular patterns (PAMPs)] activates the LP. This is facilitated by the activation of two MBL-associated serine proteases (MASP), MASP-1, and MASP-2 (8), leading to the cleavage of C4 and C2. Thus, C4 acts as the point where both the CP and LP merge, leading to the association of C4b and C2a, generating the C3 convertase (C4b2a; Figure 1).

The autohydrolysis of C3 to form C3(H_2_O) by a mechanism known as the “tick-over” helps maintain basal levels of AP. C3(H_2_O) thus formed further binds to factor B (FB) and factor D (FD). FD, being a serine protease, cleaves FB into Ba and Bb, resulting in the formation of C3(H_2_O)Bb, which, like C4b2a, cleaves C3 to form C3b and C3a. The nascent C3b formed can associate with FB and, in the presence of FD, can form C3bBb, the AP C3 convertase (9). Properdin is an important component of the AP and functions by stabilizing C3bBb and increasing its half-life by 5- to 10-fold, facilitating the further cleavage of C3 into C3b (10, 11). The C3 convertase formed by all the three pathways cleaves C3 into C3a, an anaphylatoxin, and C3b, a major opsonin. The nascent C3b is central to further amplification of the pathway and is a critical component in assembling C5 convertase of the CP/LP (C4b2a3b) and the AP (C3bBb3b), essential to cleave C5 into C5a and C5b. The C5b thus formed associates with complement components C6, C7, and C8 and multiple units of C9, resulting in the formation of a multi-protein lytic complex called the membrane-attack complex (C5b-9, MAC). Apart from the membrane lytic action of complement via the MAC, CS has a wide range of other functions. Activation products, C3a, and C5a are potent anaphylatoxins, and the opsonins C3b and C4b facilitate the engagement of innate and adaptive immune cells, playing critical roles in inducing both T- and B-cell responses (12). Active cross-talk between complement proteins and various components of the adaptive arm demonstrate the role of the CS as a connecting link between the innate and the adaptive immune system (13).

Despite the decisive roles played by the CS in host immunity, the lack in memory response and its inability to distinguish between the “self” and “non-self,” especially during unprecedented activation of complement, can be damaging to the host. This is largely checked by a highly concerted group of proteins called the regulators of complement activation (RCAs) (14). These proteins have unique domains called the complement control protein (CCP) or short-consensus repeats (SCRs), composed of 60–70 amino acids with complement regulatory activities. RCAs can be either membrane-associated, e.g., CD55 (decay accelerating factor, DAF), CD46 (membrane cofactor protein, MCP), CD59, and complement receptor type I (CR1, CD35) (15), or soluble in nature, e.g., C1 inhibitor (C1INH), C4b binding protein (C4BP), and factor H (fH) (14). CD55 and CD59 are attached to the cell membrane by a glycosylphosphatidylinositol (GPI) anchor while CD46 and CD35 are transmembrane proteins. CD55 has an inherent decay accelerating activity (DAA) and mediates the rapid dissociation of the C3 and C5 convertases, while CD59 prevents MAC formation by blocking the incorporation of C9 into the cell membrane. Factor H, CD35, and CD46, on the other hand, act as cofactors for the factor I (fI)-mediated inactivation of C3b into iC3b. Among the CP specific RCAs, C1INH binds to C1r and C1s and prevents their auto activation, while C4BP acts as a cofactor for the fI-mediated inactivation of C4b into C4c and C4d. CD35 is a highly potent and multifunctional RCA, since it exhibits both decay accelerating and cofactor activities. Not only does CD35 mediate the inactivation of C3b into iC3b, but it also supports an additional cleavage of iC3b into C3c and C3dg and acts as a cofactor for the inactivation of C4b by fI.

Investigation into virus-complement interactions had garnered much interest in the past, and this interest has only grown over the years to better comprehend the nature of these interactions. The knowledge acquired has enriched our insight into not only viral pathogenesis but also the underlying mechanisms of host immunity to these pathogens. Among animal viruses, RNA viruses constitute a large group of both veterinary and clinical significance. Based on the characteristics of the genetic material, they are classified into single- or double-stranded RNA viruses of positive or negative strand polarity that is either segmented or non-segmented. Human mortality of unprecedented proportions have been brought about by RNA viruses, the well documented being the pandemic H1N1 outbreak of 1918 (Spanish flu) that killed humans in millions (16). More recent outbreaks causing widespread devastation have been attributed to RNA viruses including Ebola, Nipah, and the ongoing severe acute respiratory syndrome coronavirus-2 (SARS-CoV-2). The nature of interaction of RNA viruses with the CS suggests a constant tussle for supremacy. This review focuses specifically on the effect of complement on RNA viruses, its role in disease severity, and the ingenious evasion strategies adopted by these viruses to counter complement.

## Complement Activation and Its Effects on RNA Viruses

RNA viruses have limited genome yet are pathogenic enough to cause devastating infections in both humans and animals. The CS that acts as a frontline defense against several pathogens is a potent barrier faced by RNA viruses (Table 1). Many characteristic features, including the molecular signatures present on the surface of RNA viruses and the presence or absence of envelope and neutralizing/non-neutralizing antibodies, contribute to the nature of interaction of these viruses with the CS. Even among the enveloped RNA viruses, the surface glycoproteins contribute significantly to complement activation.

**TABLE 1:** Complement activation and the effect of activation on RNA viruses.

Virus family	Pathway activated	Effect of complement activation
***Orthomyxoviridae***		
WS/33H1N1influenza Influenza A/Ibaraki/1/90 (H3N2) Influenza A(H1N1) pdm 009 Influenza A/PR/8/34	CP and AP LP CP and AP CP	Ab – dependent, CP- mediated virus neutralization Virus neutralization Ab – dependent, C-mediated neutralization C-deposition followed by aggregation
***Paramyxoviridae***		
Nipah virus Mumps virus Parainfluenza virus 5 Parainfluenza virus 3 Measles virus Newcastle disease virus (NDV)	CP (Ab-dependent) and AP AP AP CP (Ab -independent) AP CP, LP, and AP	Resistant to neutralization Neutralization by virolysis Neutralization by virus aggregation Neutralization by virolysis Lysis of infected cells Virus neutralization
***Pneumoviridae***		
Human respiratory syncytial Virus (hRSV)	CP and AP	Ab-dependent, C-mediated, cytolysis
***Filoviridae***		
Ebola virus (Pseudotype) Marburg virus(Pseudotype)	LP LP	Neutralization Neutralization
***Flaviviridae***		
Hepatitis C virus Dengue Virus West Nile Virus Zika Virus Japanese Encephalitis Virus	LP LP AP, CP and LP CP CP	Neutralization Ab – dependent C-mediated Cytolysis Neutralization by virolysis, block in virus fusion Neutralization by virolysis C- mediated cytolysis
***Togaviridae***		
Ross River virus Chikungunya virus Sindbis Virus	CP (Ab – dependent) —————— CP and AP (Ab – independent)	————– Opsonization NOT leading to neutralization Neutralization
***Rhabdoviridae***		
Vesicular Stomatitis virus Chandipura virus	CP CP	Neutralization by aggregation followed by lysis Neutralization by virus aggregation following opsonization
***Retroviridae***		
Human immunodeficiency virus 1	CP, LP and AP	Virolysis

*Virus families with select viruses indicating the specific pathway activated and the mechanism of neutralization. Key: CP, Classical pathway, AP, Alternative pathway, LP, Lectin pathway, C, Complement, and Ab – Antibody.*

Orthomyxoviruses are enveloped viruses and the two major surface proteins hemagglutinin (HA) and neuraminidase (NA) activate complement to varying degrees, with HA being the better activator (17). Normal human serum (NHS) could readily neutralize strain A/WS/1933 H1N1 influenza virus in an antibody-dependent manner, and this neutralization was dependent on C1, C3, and C4 and thereby the CP (18). Activation of AP by this strain required antibodies and properdin stabilization, resulting in the deposition of C3. Interestingly, neither AP nor the terminal pathway components aided in virus neutralization (19). In the case of influenza viruses, the glycosylation pattern of surface proteins is a critical factor determining the pathway activated and the degree of neutralization. Studies with a series of H3N2 and H1N1 mutants generated by swapping the HA and NA showed that those viruses with HA containing glycans rich in mannose oligosaccharides readily bound MBL and activated complement, leading to enhanced neutralization (20). The CP or LP activation by influenza viruses usually resulted in virus neutralization, which was enhanced many folds in the presence of binding antibodies (19). Direct binding of C1q to influenza B virus and the binding of MBL to the virus-infected cells facilitated active neutralization of the virus and lysis of infected cells by Guinea pig serum (17, 20). MBL could directly bind to influenza A/Ibaraki/1/90 (H3N2) and neutralize the virus and prevent the spread of virus in culture. The inhibition was due to the interaction of MBL with HA and NA (21). The complement-mediated neutralization of the PR8 strain of influenza A virus (influenza A virus A/PR/8/34) by mouse serum required the presence of natural IgM and the mechanism of neutralization was found to be the aggregation of the virus following complement deposition. The role of IgM was confirmed by using serum from Rag^–/–^ mice (18). The role of individual pathways of complement in limiting influenza A(H1N1)pdm09 was demonstrated by the use of C3^–/–^, C4^–/–^, and FB^–/–^ mice. Mice deficient in C3 were 100% susceptible; however, those deficient in C4 and FB were less susceptible, suggesting cross-talk between CP and AP. Simultaneous activation of both CP and AP followed by amplification through the AP enhanced the neutralization of the pandemic strain. The degree of neutralization correlated with the degree of C3b deposition and other complement components generated. Significant deposition of C3b observed on the surface of the seasonal influenza A (H3N2) compared to the pandemic virus suggest the preference of C3b for certain carbohydrates and its specificity to certain binding sites on them. The alteration in the C3b acceptor site on the surface glycoproteins, HA, and NA on the pandemic virus requires IgG to mediate the binding of C3b on them. Antagonists of C3a and C5a, and the corresponding receptor knock-out mice, showed that C5a played a less significant role in limiting the influenza A(H1N1)pdm09 virus infection (22).

Paramyxoviruses are negative-sense, single-stranded RNA viruses known to cause a number of important human diseases including mumps, measles, and the deadly Nipah encephalitis. Nipah (NiV) and Hendra (HeV) viruses belonging to the genus *Henipavirus* of the *Paramyxoviridae* family are deadly BSL4 pathogens. Nipah virus has two major glycoproteins, the fusion protein (F), and the glycoprotein (G). Using VSV-based pseudoviruses expressing F and G glycoproteins, it was demonstrated that these glycoproteins activate the AP. However, when the pseudotypes were primed with specific antibodies, and more interestingly with the soluble EphrinB2 (the cognate receptor for NiV) linked to the Fc region of human IgG1, the CP was found to be predominantly activated (23). Among other paramyxoviruses, parainfluenza virus 5 (PIV5), and mumps virus (MuV) were found to activate the AP, while parainfluenza virus 3 (PIV3) activated CP independent of antibodies (24–28). Natural antibodies present in the NHS was found to bind to PIV5, yet CP was not activated; however, a dramatic switch from AP to CP was noticed when a single amino acid change, G3A, was engineered in the F protein (29). This clearly suggests the importance of surface glycoproteins in determining the pathway activated. Besides complement activation, the interaction of surface glycoproteins with complement proteins is critical in determining the mechanism of neutralization. The overall structure and conformation of the viral proteins and more specifically key epitopes are important factors determining the sensitivity of the virus to specific complement pathways. Although the closely related PIV5 and MuV both activated the AP, the mechanism of neutralization is quite distinct; while PIV5 aggregated in the presence of complement, significant lysis was observed in the case of MuV (27). Newcastle disease virus (NDV), an avian paramyxovirus, was found to activate complement and the neutralization was dependent on all the three pathways. C3, C4, and C5 were found to be critical components for virus neutralization (30). Apart from the importance of the complement components, the cells in which the virus is cultured and the complement source also dictated the extent of neutralization. Egg-grown NDV was readily neutralized by human complement while primate cell derived NDV resisted neutralization (30, 31). Measles virus was found to be a potent activator of the AP, where the fusion protein (F) and not the hemagglutinin protein (HA) contributed significantly toward this activation (25). Measles virus-infected cells sensitized with antibodies against the F and HA, when exposed to C4-deficient serum, were lysed, suggesting a role for the AP (26). An antibody-independent activation of AP by measles virus-infected cells has also been reported (32). Human respiratory syncytial virus (hRSV) is an enveloped virus and a member of the family *Pneumoviridae* known to cause respiratory infections in children. Deposition of antibodies IgG, IgA, IgM, and IgE bound to hRSV antigens in the nasopharyngeal cells of children with acute hRSV infection suggested a role of complement activation in hRSV infection (33). An antibody-dependent protection in hRSV infection was later confirmed in complement-deficient mice (34, 35). HeLa cells infected with hRSV activated both CP and AP; however, anti-hRSV antibodies were required for cytolysis by complement (36, 37). The importance of pulmonary macrophages and the CS in restricting hRSV replication suggests the significance of local complement production in limiting RNA viruses (34).

Flaviviruses are a family of positive-sense, single-stranded RNA viruses primarily transmitted by ticks and mosquitoes with notable exception like the hepatitis C virus (HCV). This family of viruses is distributed globally and can cause a wide range of human illnesses including dengue hemorrhagic fever (DHF), yellow fever, and West Nile encephalitis. The LP plays a critical role in the neutralization of HCV, wherein MBL binds directly to the E1 and E2 surface glycoprotein and activate LP by engaging MASP-2 (38). The NS1 protein of dengue virus (DENV) plays an important role in complement activation. It is secreted by the infected cells and accumulates in the serum of the infected patients and can also bind back to the infected cells. The NS1 protein of dengue virus can activate complement in an antibody-independent manner; however, engagement of anti-NS1 antibody with cell surface-bound NS1 further accentuates complement activation leading to cytolysis (39–41). A key feature in DENV pathogenesis is antibody-dependent enhancement (ADE) of infectivity. Low-avidity antibodies arising from a prior exposure to a specific DENV can cross-react with other DENV serotypes during a subsequent infection, resulting in ADE and disease severity. The binding of C1q to human anti-*flavivirus* antibodies results in marked reduction of ADE in an IgG subclass-dependent manner (42, 43). Among the pool of antibodies against Japanese encephalitis virus (JEV) surface proteins in the convalescent sera of patients in JEV endemic areas, only anti-NS1 antibodies supported complement-dependent cytolysis of infected cells (44). West Nile virus (WNV) transmitted mainly through the bites of infected mosquitoes can cause fatal neurological disease in humans. The role of complement in restricting WNV infectivity was also demonstrated *in vivo*. Compared to wtC57BL6 mice, C3^–/–^, CR1^–/–^, and CR2^–/–^ mice were highly susceptible to WNV infection. Virus neutralization by complement was much enhanced in the presence of an antibody against the envelope protein (E) of WNV (45). The neutralization of WNV by serum complement was antibody dependent nevertheless; MBL was also found to directly bind to the virus, promoting opsonization by complement components and limiting virus entry (45, 46). While binding of C1q directly to the E protein of DENV could reduce virus infectivity, it was found to enhance the WNV neutralization potential of anti-WNV antibodies even at a low antibody titer (47–49). Recognition of N-linked glycans on the surface proteins of both WNV and DENV by MBL activated the LP, resulting in virus neutralization by blocking virus fusion (50, 51). Interestingly, components of all three complement pathways are essential for limiting the WNV infection (45, 50, 52). Using factor B^–/–^, factor D^–/–^, C1q^–/–^, and C4^–/–^ mice, it was clearly demonstrated that deficiency in AP components contributed significantly to neuropathogenesis, while deficiency in CP/LP components drastically affected the adaptive responses required to restrict WNV infection (52). While the neutralization of WNV and DENV by complement showed marked dependency on MBL, a less important role for the terminal pathway complement components including C5 was observed in the case of WNV (51, 53). Among the flaviviruses, the interaction of E protein–antibody complex with complement components plays a critical role in the reduction of infectivity. Neutralization of Zika virus (ZIKV), another mosquito-borne flavivirus, associated with microencephaly in the neonates and Guillain–Barré syndrome in the adults was largely dependent on CP. Natural IgM antibodies were found to have a protective role against ZIKV. The binding of C1q to both envelope (E) and NS1 proteins followed by MAC formation resulted in virolysis (54).

The *Togaviridae* is a family of enveloped, positive-sense single-stranded RNA viruses distributed globally. This family consists of two genera, namely, *Alphavirus*, and *Rubivirus*. Extensive studies on Sindbis virus of the *Alphavirus* genus showed that while the virus was capable of activating both the AP and CP, the degree of activation of AP depended on the levels of sialic acid, with low levels being a better activator of AP (55, 56). Other alphaviruses investigated for their interactions with the CS include the Ross River virus (RRV) and Chikungunya virus (CHIKV), wherein the role of complement was rather destructive to the host than protective during virus infection. CHIKV was also found to activate complement, resulting in the deposition of complement components C3b and C4b on the surface of the virus (57).

Rhabdoviruses are negative-sense, single-stranded RNA viruses with broad tropism ranging from plants to mammals. Members of the *vesiculovirus*, *lyssavirus*, and *ephemerovirus* genera of the *Rhabdoviridae* family are known to cause infection in mammals. Vesicular stomatitis virus (VSV), a prototypic member of the genus vesiculovirus, was found to activate the CP of complement either in an antibody-dependent or -independent manner. Natural IgM present in the serum was found to enhance the complement-mediated neutralization by opsonization (58, 59). Activation of complement by VSV results in initial aggregation of the virus followed by virolysis (60). Chandipura virus (CHPV), another vesiculovirus and a potent human pathogen, has also been shown to activate the CP, which involved the direct binding of C1q to the virus. This virus was highly sensitive to complement and was neutralized effectively by complement-dependent virus aggregation (61). Although VSV and CHPV belong to the same genus with a single glycoprotein (G) in the virus envelope, marked differences were observed in the mechanism of neutralization. This suggests that the interaction of viruses even within the same genus can be quite complex, thus requiring extensive investigation. Filoviruses are negative-sense, single-stranded RNA viruses with characteristic filamentous virus particles. This family of viruses includes the highly pathogenic Marburg and Ebola (EBOV) viruses known to cause fatal hemorrhagic fever. The EBOV and Marburg virus glycoproteins were found to activate the LP; however, the binding of MBL to EBOV had a mixed effect of either promoting LP progression or promoting virus entry (62).

Human immunodeficiency virus (HIV), an enveloped retrovirus, is known to activate the CP and LP independent of antibody, with direct binding of C1q to gp41 acting as a trigger for CP activation (63–66). Interaction of gp120 of HIV with the components of the CP, LP, and AP further induces complement activation. Interestingly, the CP was activated by HIV in the sera of rabbit, mouse, and guinea pig, resulting in the lysis of both virus and virus-infected cells (67–69). Enhanced activation of CP by antibodies against HIV followed by opsonization with C3 occurs during seroconversion and after transition to chronic phase (70). Human T-cell leukemia virus type 1 (HTLV-1) binds directly to C1q via its extramembrane region of the envelope protein gp21. However, binding of C1q to the cell-free virion not only inhibited its potency to infect cells but also resisted lysis by human serum. Treatment with anti C1q antibody could, however, reverse the inhibitory effect (71).

As discussed above, complement activation by many RNA viruses results in their neutralization; however, there have been exceptions to this rule. In the case of NiV, the presence of the F and G glycoproteins was sufficient to protect the VSV pseudotypes from being neutralized by complement (23). This resistance was further confirmed with wild-type NiV, which, upon exposure to complement, was found to be completely resistant to neutralization with marked reduction in surface deposition of C3b in comparison to PIV5 (72). Interestingly, similar observations were made during our investigations with CHIKV, wherein the virus was found to resist complement-mediated neutralization. Complement activation by CHIKV did result in deposition of C3b and C4b on the virus, but to a lesser degree (57). The inherent resistance to complement observed in the case of both CHIKV and NiV alludes to a virus-associated mechanism for complement resistance discussed in much detail later. Earlier *in vitro* studies demonstrated that RRV could not independently activate the AP or CP (73). However, the role of C3 in RRV pathogenesis was established when it was observed that the disease associated with RRV infection was severe in wtC57BL6 mice compared to C3^–/–^ mice (74). Extensive studies had shown that the binding of MBL to the glycans associated with the E2 glycoprotein on the RRV-infected cells enhanced the disease severity in the host (75, 76).

The outbreak of the novel SARS-CoV-2 has had a devastating effect globally. Human coronaviruses belong to the family *Coronaviridae* and primarily affect the respiratory and gastrointestinal tract of a range of animals including mammals and birds. Clinical studies based on the 2003 SARS-CoV outbreak have highlighted the significance of LP in limiting the infection. The direct binding of the spike protein (S) of SARS-CoV to the carbohydrate recognition domain of MBL resulted in complement activation and C4 deposition on virus-infected cells. The residue on the S protein that binds to MBL was identified as the N-linked glycosylation at N330 (77). This residue is close to the angiotensin-converting enzyme 2 (ACE-2) receptor binding site on the virus. The MBL-S protein interaction did not affect SARS-CoV binding to ACE-2 but blocked the binding of the virus to C-type lectin (DC-SIGN) (78). Therefore, a direct correlation of MBL versus susceptibility to virus could be drawn as most patients with SARS had MBL levels in serum significantly lower than the controls. Proteomic analysis of serum proteins from SARS patients showed significantly higher levels of C3c compared to the control sera, suggesting complement activation during SARS-CoV infection (79). Further, the N protein of SARS-CoV2 was found to activate MASP-2, leading to the aberrant complement activation and deposition of MASP-2 and C4 in the lung tissue of severely affected COVID-19 patients (80). In another study, extensive deposition of C5b-9, C4d, and MASP-2 was observed in the postmortem lungs of COVID-19 patients (81). These findings suggest that the deposition of C3 and C4 on the SARS-CoV-2 virion may lead to their neutralization (82).

## Complement: A Driver of Disease Severity

Complement, thus, is capable of restricting virus infection to a great extent. In certain viral infections, overwhelming immune response often results in significant damage to the host. Hyper-activation of complement has also been attributed to disease severity in a number of viral infections. This is well demonstrated in the case of RRV, an arthritogenic alphavirus known to cause marked inflammation of the joints. Exacerbation in the disease severity was observed in wtC57BL6 mice infected with RRV compared to C3^–/–^ mice, despite marked similarities in the level of immune cell infiltration. This clearly demonstrated the importance of C3 in promoting disease rather than limiting the virus spread (74). Further studies demonstrated that the severity in the arthritic disease is dependent on complement receptor 3 (CR3 or CD11b/CD18). CR3^–/–^ mice had only mild disease symptoms; however, similar to the C3^–/–^ mice, the absence of CR3 did not affect recruitment of cellular infiltrate at the localized sites of inflammation. Therefore, the CR3-dependent signaling that is required for the overall expression of proinflammatory molecules was found to be the contributing factor for disease severity (83). In addition to C3, MBL and hence the LP contributed to the disease severity in mouse models of RRV infection, as increased deposition of MBL in the muscle tissue of RRV infected wtC57BL6 compared to MBL^–/–^ mouse correlated with the enhanced disease severity in the former. Interestingly, the severity of RRV-induced symptoms in C1q^–/–^ and Factor B^–/–^ mice were comparable to wild type, suggesting the decisive role of LP in the disease prognosis. The glycans on the E2 protein of RRV was found to be the recognition sites for MBL, as mice infected with mutant RRV lacking the glycans had less pronounced symptoms despite both the viral load and the infiltration of proinflammatory cells being comparable to mice infected with wild-type RRV (75). These findings are clinically relevant as it was also demonstrated in the same study that human patients with RRV infections had substantially higher concentrations of MBL in both serum and synovial fluids compared to that of healthy individuals (75). Hence, it is clear that complement contributes significantly to the overall disease severity during RRV infection.

The role of complement in disease enhancement has also been well documented in flaviviral infection. In severe forms of DENV infection including DHF and dengue shock syndrome (DSS), complement activation caused significant damage to the host. Complement activation markers C3a and C5a (potent anaphylatoxins) were found to promote vascular leakage, contributing directly to DSS (52, 84–89). This is further supported by the findings that prior to enhanced consumption and reduction in complement components in the plasma of DSS patients, a significant increase in C3a and C5a was observed (87, 90–92). Other key observations supporting a role for complement in the severity of DENV disease include the activation of complement followed by MAC formation in the presence of anti-DENV antibodies; enhanced levels of C5a, C3a, NS1, and soluble C5b-9 in DHF patients; and, more indirectly, the observation that CD59 was found to be up regulated in patients with less severe form of disease (39, 93, 94). The relevance of complement in disease exacerbation during WNV infection is well documented clinically as well as in mouse models. *In vitro* studies have demonstrated that complement supported WNV infection with increased virus output, which required the cross-talk between IgM and CR3. In the absence of CR3, the effect was found to be reversed (95). The memory impairment due to the elimination of presynaptic terminals, observed in the survivors of West Nile encephalitis, was attributed to complement with C3 playing a major role. Up regulation of C1q in infected neurons and microglia and also the presynaptic terminals in mouse models further validated the damaging effect of complement in WNV infection (96).

Human immunodeficiency virus-associated neurocognitive disorder (HAND) can be due to either HIV encephalitis (HIVE) or mild neurocognitive impairment (NCI), for which the neuropathological substrate has not been established. Earlier studies have suggested that the central nervous system (CNS) dysfunction in HIV infection is due to indirect effects rather than neuronal or glial infection (97). The expression of complement components including C3 was significantly higher in brain tissues of HAND patients, and marked C3 reactivity was observed in astrocytes and neurons. Exposure of human fetal astrocytes to HIV in culture induced C3 promoter activity, mRNA expression, and protein production via NF-κB and protein kinase signaling in an IL-6-dependent manner (98). Similarly, when the global gene expression profile of various brain regions from HIV-1-infected patients without substantial NCI, those with substantial NCI without HIVE, and those with substantial NCI with HIVE against uninfected controls was performed, not only was HIV-1 RNA found to be substantially higher [3log (10) units], but several complement components were significantly up regulated in the neostriatum of NCI with HIVE group. Thus, the CS has a potential role in the overall pathogenesis of HIVE-induced NCI (99). Exposure of primary astrocytes and different cell lines of astrocytic origin to HIV-1 resulted in marked up regulation of C2 and C3 expression. The up regulation of C3 transcript and protein involved HIV-1 co-receptor engagement by both laboratory and primary isolates of HIV-1. Most importantly, the C3 induced was biologically active in the complement cascade (100). These findings hold huge significance as elevated levels of C3 and C4 in the CSF of HIV-infected individuals have been reported (101). In addition to this, significant up regulation in the cerebral synthesis of complement factors, C1q and C3, was observed in a range of cell types including astrocytes, neurons, microglia, infiltrating macrophages, and multinuclear giant cells in monkeys challenged with simian immunodeficiency virus (SIV) compared to the control animals. Up regulation also led to the enhanced deposition of both C3 and C1q on neighboring neuronal cells leading to lysis (102). This could possibly account for the cellular damage in brain observed during neuro-AIDS. Peripheral blood T-lymphocytes from HIV-infected patients had decreased levels of CD59, suggesting suppression of CD59 expression by HIV (103). Upon exposure of human neuronal (SK-N-SH) and astrocyte (T98G) cell lines to either recombinant HIV gp41 protein or an immunodominant gp41 peptide, significant reduction in CD59 mRNA and protein was observed (104). These findings further substantiate that not only CD59 but also other proteins of the complement activation pathway are modulated by HIV. This, in turn, may contribute to the neuronal and glial cell damage in HAND patients.

Persistent activation of complement is one of the causative factors in chronic HCV infections, which may further lead to liver fibrosis (105). Contribution of C5 in mediating HCV-associated hepatic fibrosis was elucidated *in vivo*, and treatment with small-molecule inhibitors against C5aR exhibited antifibrotic effects (105). Several experimental and clinical studies so far support the involvement of complement dysregulation in intra- and extrahepatic disorders like type II mixed cryoglobulinemia (MC) and B cell lymphoma (106). A higher prevalence of anti-C1q antibodies among HCV genotype IV-infected patients with extrahepatic autoimmune involvement has been reported. HCV patients with immune complex diseases like systemic lupus erythematosus, cryoglobulinemia, glomerulonephritis, vasculitis, and severe rheumatoid arthritis had significantly higher levels of anti-C1q antibodies, which correlated to decreased C4 levels (107, 108). Hence, sustained activation of complement or suppression of complement by the core protein of HCV added to the significantly high levels of anti-C1q antibodies support manifestation of HCV-associated liver diseases.

More recently, MASP-2 has been implicated as one of the factors contributing to aggravated lung injury in COVID-19 pathogenesis. It was demonstrated that the N-protein, a secreted protein of SARS-CoV, MERS-CoV, and SARS-CoV-2, binds to MASP-2 (80). The binding site for MASP-2 on the N-protein of SARS-CoV was identified to be between the amino acid residues 116–124 in the coil motif of the N-protein. Binding of MASP-2 to the N-proteins of SARS-CoV-2 and MERS-CoV was traced to N (115–123) and N (104–112) regions that shared a high level of identity with the SARS-CoV N (116–124) motif. The N-proteins of all three viruses potentiated MBL–MASP-2 interaction, resulting in aberrant complement activation and subsequent deposition of C3 and C4 component. Using an adenovirus system encoding SARS-CoV-N and MERS-CoV-N, the investigators could further substantiate the role of N protein in acute inflammation. Administration of C1INH or an antibody against MASP-2 could substantially limit the damage. These findings were further validated using MASP-2 knockout mice, wherein the disease symptoms post N-protein challenge were less severe with longer survival rates than that of the wild-type mice, suggesting the importance of MASP-2 in SARS infection (80). The immunohistochemical analysis of postmortem lung tissues from five COVID-19 patients who died of respiratory failure showed enhanced deposition of MBL, MASP-2, and other markers including C4d and C5b-9 co-localizing with S protein of SARS-CoV-2 (80, 81). The serum samples also had elevated levels of C5a, suggesting hyperactivation of complement systemically or as a result of leakage of activated fragments from diseased lung (82). SARS-CoV-2-infected A549 cells showed differential expression of C3, C1r, and other complement proteins. Serum C3 levels were reduced in ∼57% of individuals, suggesting C3 consumption followed by complement activation (109). Of much interest was the finding that the anti-C5a antibody (BDB-001) had much promise in the recovery of severe COVID-19 patients (80, 82). Thus, the overall effect of RNA virus-complement interaction can be multifaceted as these interactions can either limit or promote the severity of disease.

## Complement at the Crossroads of Innate and Adaptive Immunity in RNA Virus Infection

A growing body of investigation reveals that the delicate balance between complement activation and regulation is one focal point of the disease outcome in viral infections. Apart from the immense role it plays in innate immunity, ample evidence points to the existence of cross-talk between the CS and the adaptive immune system.

Increased levels of C3a and C5a in the bronchoalveolar lavage fluid (BALF) and serum from patients infected with H1N1 pandemic virus suggest their role in virus-induced acute lung injury (ALI). The increased expression of C5aR on dendritic cells (DCs) promoted CD8^+^ T cell activation by C5a. Mice treated with C5aR-specific antagonist showed a reduction in the flu virus-specific CD8^+^ T cells accompanied by attenuated cytolysis in the lung (110). C5a-mediated leucopenia, reduced antigen-presenting cells, and impaired T cell responses had been observed in severe H7N9 influenza infection (111, 112). C5a being a chemoattractant for neutrophils and monocytes activates these cells to generate reactive oxygen species (ROS) (113). Studies from mice infected with influenza suggested a critical role of ROS in infection-induced pneumonia (114). Treatment with antioxidants could effectively reduce the lung damage and mortality in these mice (115). Increased expression of C5aR1 in bronchial epithelial and inflammatory cells accompanied by increase in C5a levels and deposition of C3 and C5b-9 in patients points to the association of C5a-C5R1 signaling in the systemic inflammatory response and the local tissue damage. Blockade of C5aR1 restricted viral replication in lung tissue and also alleviated ALI (116). The C3d and C3a generated during influenza A(H1N1)pdm09 infection plays a crucial role in evoking B cell as well as CD4^+^ and CD8^+^ T cell responses, which helps to contain the virus infection (22). The role of MBL in up regulating inflammatory responses causing tissue damage was demonstrated in MBL knockout (KO) mice challenged with the pandemic H1N1 or the avian influenza H9N2/G1. The KO mice developed less severe disease as indicated by the reduced weight loss when compared to the wild type. Increased levels of proinflammatory cytokines and chemokines in the wild-type mice were also observed when compared to MBL KO mice, suggesting a role of MBL as a contributing factor to disease severity (117).

Increased viral load in the brain of mice infected with WNV, in the absence of C3 or CR1/CR2, suggested their importance in limiting viral infection. Deficiency in the components of the CP (C4 and C1q) was associated with decreased splenic infections and impaired B and T cell responses to WNV infection as C4 generated anti-WNV IgM responses, while C4 and C1q were required for anti-WNV IgG responses (45). In mouse macrophages, a CR3 mediated complement-dependent enhancement of WNV infection was observed.

Hepatitis C virus is known to cause persistent infections in individuals which later on may lead to chronic liver diseases such as cirrhosis and hepatocellular carcinoma. Defective expansion and differentiation of HCV-specific T cells observed in chronic HCV patients suggest an impaired antiviral immune response during the early stages of infection. A role of HCV core protein in immunomodulatory functions was demonstrated in chronic HCV patients and *in vivo* (118). The interaction of HCV core protein with gC1q receptor on human monocyte-derived DCs inhibited TLR-induced IL-12 production alone and not any other cytokines induced by TLR (119). Moreover, the engagement of core protein with gCIqR on the DCs also inhibited the production of IL-12 and mediated the production of Th2-induced cytokines such as IL-4 upon co-culturing with CD4^+^ T cells. Patients with chronic HCV infection showed persistent expression of gC1qR^+^ CD4^+^ T cells, impairing T cell responses (120). These results taken together suggest that HCV limits the induction of Th1 response through the binding of core protein to gC1qR on the DCs, contributing to viral persistence (119).

The binding of HIV-1 gp41 to gC1qR on uninfected CD4^+^ T lymphocytes resulted in NK cell-mediated lysis of these cells during HIV infection (121). HIV, during its dissemination through the bloodstream, is opsonized and adheres to the erythrocyte membrane through CR1. The presence of antibodies enhances this binding (122). The absence of complement opsonins on HIV or the blockade of CR1 inhibited virus adherence to RBCs. The CR1 inactivates C3b into iC3b and C3d. The C3d-bound HIV-1 is then trapped in follicular dendritic cells (FDCs), where it remains infectious and intact, acting as a reservoir (123). Opsonized HIV can also bind to B cells expressing CR-2 and further results in B-cell-mediated transmission of the virion to HIV-negative CD4^+^ T cells in a CR1- or CR2-dependent manner (124). CR3 also plays an important role in HIV infection via modulating TLR 8-mediated signal transduction (125). Opsonization of retroviral particles with complement proteins has been shown to enhance the ability of DCs to induce the activation, expansion, and differentiation of virus-specific cytotoxic T lymphocyte (CTL) responses, both *in vitro* and *in vivo* (126). Exposure of DCs to C-opsonized HIV was able to activate DCs, which, in turn, stimulated CTLs to elicit antiviral activity, significantly better than non-opsonized HIV (126). The presence of HIV-specific CTLs correlated with the decline in viremia during the acute phase, but not during the chronic phase of infection (127, 128). The susceptibility of monocyte-derived macrophages (MDM) to HIV-1 infection was enhanced multiple folds upon pre-exposure to C5a or C5a^*desArg*^. This was attributed to a notable increase in the secretion of TNF- α and IL-6, suggesting the role of complement anaphylatoxin in inducing proinflammatory responses, thereby supporting virus infection (129). Thus, C5a-elicited proinflammatory cytokines and recruited DCs can indirectly promote infection of MDMs and T cells (129, 130). HIV-complement interaction results in the generation of C5a, which, besides its anaphylactic property, can exert its function by interacting with C5a receptor 1 (C5aR1; CD88) and C5a receptor 2 (C5aR2, C5L2). In myeloid cells, C5aR1 heterodimerization with CCR5, a co-receptor for HIV, was observed. C5a receptor-specific antagonist or neutralizing monoclonal antibodies against C5aR1 could limit the integration of the R5 strain of HIV in MDM by reducing the CCR5 expression. Since the levels of C5aR1 in MDM of CCR5D32 homozygous individuals were comparable to that of the normal controls, it is evident that C5aR1 works in concert with CCR5 promoting HIV entry into macrophages (131). The opsonization of HIV-1 by complement is a critical factor in driving the hyper-expression and secretion of key cytokines, transforming growth factor b (TGF β), interleukin 1b (IL-1β), interleukin 6 (IL-6), and interleukin 23 (IL-23) in DCs supporting the T helper 17 cell (TH17) induction. The opsonized HIV mediated the expression of TH17 polarizing cytokines in DCs, via the extracellular signal-related kinases (ERK) and induced C3a production in these DCs, enhancing TH17 polarization (132). The type of opsonin on HIV also determined the type of response generated in DCs. While exposure of DCs to complement-opsonized HIV supported HIV-specific CD8^+^ T cell response, HIV opsonized with IgG dampened this response (133). DCs are known to mount potent antiviral responses that can limit HIV infection, which includes the Sterile alpha motif and HD-domain-containing protein 1 (SAMHD1)-mediated response. However, complement-opsonized HIV overcomes SAMHD1 and other restrictions mounted by DCs to productively infect these cells. Interestingly, the degradation of SAMHD1 was not observed but phosphorylation of T_592_ in SAMHD1 restricted intracellular replication of HIV-1. Not only was the virus replication restricted, complement-opsonized HIV induced multiple changes in the DCs including DC maturation and expression of co-stimulatory molecules of Type I interferon genes, thereby stimulating HIV-specific CD4^+^ and CD8^+^ cells (134).

The disease progression to severe pneumonia and acute respiratory distress in SARS patients has been attributed to the hyperactivation of complement (135). The systemic activation of complement mediated by MBL in SARS-CoV-2 has also been demonstrated by the co-localization of MASP2 with the spike protein (S) in the lungs of COVID-19 patients (81). Most of the disease-associated inflammation and hypercoagulability is a direct effect of the binding of the complement component C5a to its receptor, C5aR on the T cells, activating them. This binding results in a hyper-inflammatory response called the “cytokine storm” (136). C5a also promotes immune paresis and exhaustion of antigen-specific lymphocytes in cases of higher viral load. C5a-mediated IL-6 and IL-8 release in SARS-CoV-1 and MERS-CoV infections diminishes the antigen-presenting ability of DCs to T cells. Also, declined production of antiviral cytokines like IFN-α, -β, and -γ and IL-12p40 with an up regulation in proinflammatory chemokines dictates the imbalance in the pro- and anti-inflammatory cytokines in patients with H7N9, SARS-CoV-1, MERS-CoV, and the current SARS-CoV-2 infections (137). C5a-C5aR axis thereby plays a critical role in mediating damage caused by highly pathogenic viruses.

## Complement Evasion by RNA Viruses

**FIGURE 2:**
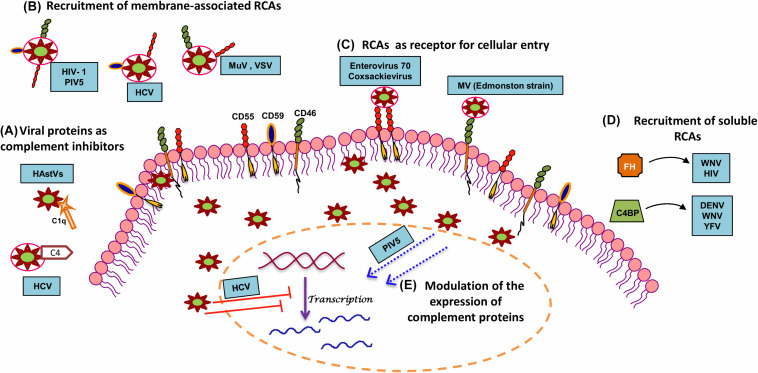
Strategies adopted by RNA viruses to modulate complement. In order to productively infect the host, RNA viruses have adopted several strategies to subvert the neutralizing effects of complement. A few mechanisms include **(A)** utilization of viral proteins as complement inhibitors, **(B)** recruitment of membrane-associated RCAs, **(C)** utilization of complement proteins for cellular entry and propagation, **(D)** recruitment of soluble RCAs, and **(E)** modulation of host complement protein expression. Examples of RNA viruses adopting these mechanisms are highlighted in blue boxes. Key: HAstV (human astroviruses), HCV (hepatitis C virus), HIV-1 (human immunodeficiency virus type 1), MuV (mumps virus), PIV5 (parainfluenza virus type 5), VSV (vesicular stomatitis virus), MV (measles virus), WNV (West Nile virus), DENV (dengue virus), YFV (yellow fever virus), and HIV (human immunodeficiency virus).

The response of complement to RNA virus infection is potent enough to restrict these viruses. It might appear that the CS is insurmountable and can, therefore, tip the balance always in favor of the host. On the contrary, these viruses have evolved to adapt and have adopted unique strategies to limit the damaging effects of complement (Figure 2 and Table 2). Again, the limited genome in the case of RNA viruses is not a handicap as these viruses coexist with the host and exploit components from the host that favor their survival. This strategy strongly works in favor of enveloped RNA viruses; nonetheless, RNA viruses that lack envelope also possess certain characteristic features that help them counter the effects of complement. The sections below discuss the strategies adopted by RNA viruses to counter the effects of complement.

**TABLE 2:** Evasion strategies adopted by RNA viruses against the complement system.

S.No.	Complement evasion strategy	Virus family and examples
1.	Recruitment of membrane-associated regulators of complement activation	***Rhabdoviridae*** Vesicular stomatitis virus (CD46, CD55) ***Paramyxoviridae*** Parainfluenza virus 5 (CD46, CD55) Newcastle disease virus (CD55, CD46) ***Flaviviridae*** Hepatitis C virus (CD59) ***Retroviridae*** Human immunodeficiency virus type 1 (CD46,CD55,CD59) Human T-cell leukemia virus type 1 (CD55,CD59)
2.	Incorporation of soluble regulators of complement activation	***Flaviviridae*** Dengue virus (C4BP, Clusterin, Vitronectin) West Nile virus (fH, C4BP) Yellow fever virus (C4BP) ***Retroviridae*** Human immunodeficiency virus type 1 (fH)
3.	Virion-associated protease activity (FI-like activity)	***Paramyxoviridae*** Nipah virus ***Togaviridae*** Chikungunya virus
4.	Exploitation of complement proteins as entry receptors	***Paramyxoviridae*** Measles virus – Edmonston strain (CD46) ***Picornaviridae*** Echoviruses (CD55) Group B coxsackieviruses (CD55)
5.	Transcriptional regulation of complement proteins	***Flaviviridae*** Hepatitis C virus (HCV down regulation of C2, C3 and C9) ***Paramyxoviridae*** Parainfluenza virus 5 (up regulation of CD59 and CD55) ***Retroviridae*** Human immunodeficiency virus type 1 (down regulation of CD59 and up regulation of C3 and C1q in the brain) ***Pneumoviridae*** Human respiratory syncytial virus (Up regulation of CD59 and CD55)
6.	Viral proteins as complement inhibitors	***Astroviridae*** Human astrovirus 1 (Coat protein binds to C1q and arrests C1s) ***Orthomyxoviridae*** Influenza virus (A/WSN/33) M1 protein binds to C1q and blocks its interaction with IgG ***Flaviviridae*** Bovine viral diarrhea virus (E2 protein) Hepatitis C virus (NS3/4A binds to C4, cleaves γ subunit)

### Viral Proteins as Complement Inhibitors

Non-enveloped RNA viruses lack the host-derived membrane but have unique mechanisms for targeting complement. Human astroviruses are icosahedral RNA viruses known to cause gastroenteritis. The direct association of the human astrovirus type 1 (HAstV-1) coat protein with C1q, an upstream component of CP, mediated the dissociation of C1s from the protease tetramer. This arrested the C1s cleavage, limiting the progression of the pathway, resulting in the decreased levels of C3b and C4b. The coat proteins of at least two other serotypes, HAstV-2 and HAstV-4, were also found to inhibit complement activation, suggesting that this inhibition was not restricted to HAstV-1 alone (138). Further investigations revealed that the inhibitory potential of the coat protein of HAstV-1 was not restricted to the CP but also included the LP by the direct binding of coat protein to MBL. The finding that the coat protein can inhibit C5a generation leading to a substantial reduction in C5b-9 holds clinical relevance as HAstV-1 infection causes gastroenteritis with a low inflammatory status in children (139). Hence, it is evident that the structural proteins in non-enveloped viruses can have direct modulatory effects on complement activation, thus limiting virus neutralization.

Proteins of enveloped viruses are also known to interact with key complement components and inhibit complement function. HCV belonging to the family *Flaviviridae* has a unique mechanism of modulating the CP. The NS 3/4A, a non-structural protein of HCV with protease activity, could directly bind and cleave the γ-subunit of C4, abrogating the effect of complement on the virus (140). The structural protein E2 in the bovine viral diarrhea virus has also been shown to block complement-mediated cell lysis, thereby acting as a potent inhibitor of complement (141). The NS1 protein secreted from the DENV-infected insect cells contains glycans rich in mannose. This secreted form of NS1 could also be identified in the saliva of DENV-infected *A. aegypti* mosquito. Independently, while DENV-NS1 could bind to C1s, C4, and C4BP, it was also found to bind to MBL, protecting the virus from neutralization by complement at the local sites of infection (142). WNV NS1 also binds to C1s and C4 and inactivate C4, thereby attenuating complement activation (143).

The matrix protein (M1) of influenza virus (A/WSN/33 strain) is an abundantly expressed protein in virus-infected cells and is released during necrosis to protect the newly formed virus particles from complement-mediated neutralization. The N-terminal domain of M1 binds to the globular head of C1q, blocking the interaction of IgG and C1q and thereby the CP (144).

### Exploitation of the Complement Pathway Proteins

The main role of proteins associated with all the three complement pathways is to target pathogens including viruses, which, upon activation, facilitate virus neutralization. In many instances, these proteins have been found to be exploited by viruses to gain entry into cells, to replicate and utilize such cells as reservoirs, and to enhance virus spread. Many viruses preferentially exploit upstream components of the CP and LP like C1q and MBL to enhance infectivity and spread. The NS1 proteins of flaviviruses WNV, DENV, and yellow fever virus (YFV) have been shown to directly interact with C4, limiting the CP and LP. The NS1 protein directly binds to C4 and recruits C1s, the protease in the C1 complex, forming the C4–NS1–C1s complex that promotes active sequestration of C4, protecting the virus from the neutralizing effects of complement (143). Interaction of HCV core protein with gC1qR was found to have major implications in the immune responses to the virus (145–147).

Mannose binding lectins were found to enhance the infectivity of a range of RNA viruses including WNV, NiV, Hendra virus, and EBOV. Using EBOV glycoprotein pseudotyped lentivirus, it has been demonstrated that specific epitopes on the N-linked glycans of EBOV glycoproteins bound specifically to the carbohydrate recognition domain on MBL. Enhancement in virus uptake and infection due to MBL binding was found to be dependent on C1QBP (gC1q receptor) (148). The binding of mucin like domain of EBOV (Zaire strain) glycoprotein to the fibrinogen-like recognition domain of ficolin-1, in the presence of limited complement, promoted virus entry rather than neutralization via the LP. The significance of this interaction is that ficolin-1 was found to enhance EBOV infection in an antibody- dependent manner (149).

### Modulation of the Expression of Complement Components

RNA viruses have also been shown to down regulate the synthesis of complement proteins. HCV effectively puts a block in the progression of the complement pathway by transcriptionally regulating expression of critical components like C3, C4, C2, and C9 (150–153). Analysis of the liver tissue from chronic HCV patients showed a reduced expression of C4 mRNA. The NS5A protein of HCV was found to modulate the expression of two of the transcription factors, namely, upstream stimulating factor I (USF-1) and inhibiting interferon regulatory factor I (IRF-1), which are essential for either basal or IFN-γ-induced C4 expression (151). Similarly, HCV was also found to modulate C3 mRNA expression. The down regulation of farnesoid X receptor (FXR) by the HCV core protein weakly represses the C3 promoter activity, whereas NS5A protein, by inhibiting the expression of CAAT/enhancer binding protein beta (C/EBP-β), a transcription factor known to bind to the IL-1/IL-6 response element in the C3 promoter, strongly repressed IL-1β-induced C3 promoter activity. Thus, active modulation of C3 transcription factors by HCV results in the underexpression of C3 in chronic HCV patients (150). Both HCV and its core protein were found to repress C9 mRNA and protein expression. The core protein modulated T cell factor-4, the transcription factor regulating C9 promoter activity, resulting in the decreased levels of C9 mRNA in liver tissue and C5b-9 in the sera of chronic HCV patients (152). Marked decrease in C2 mRNA in chronic HCV infection also contributed to significantly low levels of CP C3 convertase (C4b2a). This resulted in a reduction of overall C3 turnover and diminished C3b deposition on target cells (153). HCV NS2 and NS5B proteins are also responsible for HCV-associated decrease in MHC class I chain-related protein A and B (MICA/B), resulting in a loss of the C3/C4 complement components. This inactivation of the CS leads to impairment of NK cell activation and attenuated adaptive immune response (154). Taken together, the potency of complement is reduced during HCV infection due to the active modulation of complement gene expression at the level of transcription. Besides modulation of complement components of the activation pathway, certain RNA viruses also modulate the expression of regulatory proteins or inhibitors of complement. Paramyxoviruses PIV5 and hRSV are known to up regulate the expression of CD55 (155) and CD59 (156). The up regulation of the regulatory proteins favors the virus as it can readily incorporate these proteins into their envelope during budding, limiting the neutralizing effect of complement.

### Recruitment of Membrane-Associated Regulators

Enveloped RNA viruses acquire their envelope from the host during the process of virus egress. This, in many instances, turns out to be advantageous for the virus, as it also picks up the host proteins associated with the membrane, which can confer protection from the host complement. Widely reported host membrane-associated RCAs in the virus envelope include CD46, CD55, and CD59. RNA viruses including HIV-1, PIV5, MuV, NDV, and VSV are known to incorporate these RCAs into their envelope (30, 60, 155, 157, 158). Extensive studies on the virus-associated CD46 and CD55 show that they are functional, with CD46 acting as a cofactor for the fI-mediated cleavage of C3b into iC3b, and CD55, accelerating the decay of complement convertases protecting these viruses from complement (60, 159). HCV particles generated from hepatic cells were found to harbor CD59, an inhibitor of the terminal pathway of complement. CD59 associated with the virion conferred protection to the virus from antibody-dependent complement-mediated lysis. Addition of CD59 blockers to both cell line derived and plasma primary virions resulted in substantial virolysis in the presence of complement, which highlights the significance of CD59 in protecting HCV from complement (160).

Human immunodeficiency virus 1 incorporates CD46, CD55, and CD59 into the virus envelope during egress. The presence or absence of the RCAs in the virus envelope depended on the cell type in which the virus was generated. The levels of RCA incorporated into the virus envelope dictated the extent of neutralization. When sensitized with gp160 antibodies, only those viruses generated in cell lines underwent virolysis; however, primary HIV isolates required sensitization with anti-RCA antibodies to undergo virolysis by complement. Besides the primary isolates of HIV-1 and specific cell line-derived HIV, (SIV delta/B670) was also found to incorporate all three RCAs. The protective role of the RCAs in limiting the effect of complement on HIV is well evidenced from the findings that HIV cultured in CHO cells with individual RCAs was significantly protected from complement-mediated virolysis compared to those without the regulators (157, 158, 161–163). Human T cell leukemia/lymphoma oncovirus type I (HTLV-1) cultured in MT2 cells contained both CD55 and CD59 in the virus envelope. The incorporated RCAs protected the particles from complement-mediated neutralization. However, this was reversed when the particles were treated with anti-CD55 and anti-CD59 antibodies or phospholipase C (164). Although it could well be considered that incorporation is a random process, growing evidences as in the case of HCV, RSV, and PIV5 suggest the recruitment of CD55 and/or CD59 to be more specific in nature because the respective virus infection induced up regulation of the RCAs in infected cells (155, 156, 165, 166).

### Recruitment of Soluble Regulators

RNA viruses not only incorporate membrane bound RCAs but also recruit soluble RCAs. The NS1 protein of DENV, WNV, and YFV were all found to recruit C4BP, an RCA of the CP (167), while other complement regulators reported to bind to NS1 include fH (168), clusterin (169), and vitronectin (170). The secreted WNV NS1 protein recruits soluble fH either in solution or onto the cell surface, as NS1 is known to bind back to cell surface. The fH thus recruited acted as a cofactor for the factor I-mediated cleavage of C3b into iC3b. The fH bound to NS1 protein can rapidly regulate complement in solution and limit complement deposition and damage to the virus and virus-infected cells (168). It can also restrict both CP and LP by binding to C4BP and inactivating C4b, both on the cell surface and in solution (167). Recruitment of soluble RCAs by NS1 protein is not pan-flavivirus as it has been demonstrated that the NS1 protein of JEV was incapable of recruiting fH (44). As mentioned above, the NS1 protein of at least three flaviviruses binds to C4BP and acts as a cofactor for the inactivation of C4b into C4c and C4d both in solution and on the cell surface. The binding site of NS1 on C4BP was mapped to the α-chain of C4BP (167). The DENV NS1 protein can also interact with clusterin, an important regulator of the terminal pathway of complement. This interaction prevents the assembly of MAC and thereby, virolysis (169). The NS1 protein of flaviviruses, including DENV2, ZIKV, and WNV, has been shown to play a role in inhibiting the terminal pathway of complement by inhibiting C9 polymerization. Additionally, the NS1 protein of DENV-2 was found to bind to vitronectin, a regulator of the terminal complement pathway and block C9 polymerization and MAC assembly (170). Among the retroviruses, HIV is known to recruit fH with the binding target being the Env proteins gp120 and gp41 (171–173). Factor H was found to have a dominant role in protecting HIV from complement. This is highlighted by the finding that even in the presence of anti-HIV antibodies, HIV was not neutralized by NHS but was neutralized by fH-deficient serum. The neutralizing effect of complement in NHS could be restored only upon inhibiting the interaction of fH with gp41, using a specific blocking monoclonal antibody against the fH binding site on gp41 (173).

Most of the evasion strategies described above involved recruitment of either membrane-associated or soluble RCAs and inhibitors of complement. Factor I is an important and unique serine protease involved in the regulatory arm of the complement. Factor I mediates the inactivation of C3b into iC3b or C3c and C3d, and C4b into C4c and C4d in the presence of specific cofactors. Earlier we had discussed that NiV exhibits an fI-like activity that could inactivate C3b but not C4b. Only the first order of C3b inactivation, which is the conversion of C3b into iC3b, was supported by the NiV-associated fI-like activity. Conversion of C3b into iC3b occurred with the incubation of C3b, fH, or sCR1 and wtNiV without the addition of fI. CD46 was also found to be associated with wtNiV, but interestingly, the associated CD46 was incapable of inactivating C3b into iC3b even with the addition of fI (72). More recently, it was identified that CHIKV also has an fI-like activity that contributes to its complement resistance. However, a stark difference was that the CHIKV-associated fI-like activity supported only the fH and not CD35-mediated inactivation of C3b into iC3b. Like NiV, CHIKV-associated fI-like activity was incapable of inactivating C4b. Although electron microscopic analysis pointed to an association of fI to the CHIKV envelope, immunoblotting and function blocking cofactor activity assays with an antibody targeting fI protease activity showed that CHIKV-associated fI-like activity is not of host origin (57). Since the source of fI or fI-like activity is not yet determined at least in the case of CHIKV, detailed investigations are required to gain a better understanding on the precise mechanisms involved.

### Complement Proteins as Tools for Virus Attachment and Entry

Receptor usage is a key factor that determines the host range in many viruses. Viruses using RCAs to attach to and infect cells have been widely reported among the members of the *Picornaviridae* family. Many subtypes of echoviruses like echo-6, -7, -11, -12, -20, and -21 bind to CD55 to gain access into the cell (174) and further studies established the binding site of echo-7 virus to CCPs 2–4 of CD55 (175). In contrast, many types of coxsackieviruses and enterovirus 70 also bind to CD55, but this attachment did not directly facilitate virus entry (176–179). Group B coxsackieviruses (CVBs) binding to CD55 on the apical surface of cells resulted in the clustering of CD55. This activated Abl kinases, resulting in actin remodeling, which delivers the virus to the tight junctions. At the tight junction, it interacts with the coxsackievirus and adenovirus receptor (CAR), leading to the internalization and the subsequent uncoating and replication (180). The Edmonston strain of measles virus uses all four isoforms of CD46 as its cellular receptor binding to CCP-1 and -2 with hemagglutinin being the interacting viral surface glycoprotein (181–185). CR3 and CR4 are used by hantavirus (186) for entry, while CR4 is exploited by rotavirus (187).

## Concluding Remarks

Complement–RNA virus interactions are complex, with each remaining in the cross hairs of the other. The role of the CS is undeniably that of a barrier at all levels of virus infection, with RNA viruses facing stiff resistance from the very early stages of infection. Infection with RNA viruses mostly begins locally, involving skin (vector borne infections) or the mucosal surface. It has been well established that the CS is active at these local sites of infection (2, 3). Surface features of these viruses dictate the type of pathway activated, with some viruses even activating more than one pathway (52). These viral signatures are so critical that even slight alterations can cause switch in pathways (29) and can define the host responses to infection, which include disease severity. These initial interactions set the stage for sequential progress of the complement cascade promoting virus neutralization. Yet again, marked variations exist even among closely related viruses with regard to the mechanism of neutralization (Table 1) (27, 60, 61). Although it may be anticipated that surface glycoproteins could contribute to these differences, in-depth investigations into these interactions are required to better understand their role. Another key aspect is the damaging effect the CS imparts on the host during the process of virus clearance. Hyperactivation of complement during infection by certain members of the *Togaviridae*, *Flaviviridae*, and *Coronaviridae* plays a key role in disease severity. The cross-talk between complement and other components of the immune system during RNA virus infection plays a pivotal role in the overall pathogenesis, with the latest example being complement-mediated disease enhancement in SARS-CoV-2 infection. Thus, RNA viruses appear to be in the cross hairs of complement.

The big question that arises out of RNA virus–complement interactions is how these viruses productively infect and cause diseases in the host, overcoming complement. Both enveloped and non-enveloped RNA viruses have ingenious ways of limiting complement. Many DNA viruses including poxviruses and γ-herpes viruses have a large genome that supports expression of viral homologs that mimic the functions of complement regulatory proteins. RNA viruses might appear at a disadvantage with respect to its genome size; however, they are known to thwart complement in multiple ways. One well-studied mechanism is the recruitment of the host RCAs by enveloped RNA viruses. The protective effect of these RCAs on the virus is very significant, especially at the local sites of infection. Another area requiring significant investigation is the nature of incorporation of the RCAs. Detailed investigation is required to further understand the specificity or selectivity of incorporation of these RCAs.

The evasion strategies adopted by RNA viruses bring the CS in the cross hairs of the virus. It is clear that the balance is quite delicate and can tip to any side, as is evidenced in the case of flaviviruses like DENV and more recently in SARS-CoV-2. Approaches to combat SARS-CoV-2, although not proven completely, include targeting complement activation to better manage the disease symptoms. Exploitation of RNA viruses as vaccine and oncolytic vectors is an emerging field. Viral vectors should be attenuated enough so as to not cause disease but yet be capable of productively infecting the target cells. An attenuated vector harboring RCAs would be a better candidate to bypass complement yet meet the requirements. Thus, a rational approach to develop therapeutics, inhibitors, or viral vectors should also take the role of complement into consideration. In conclusion, studies have highlighted the complexities in RNA virus–complement interactions, but with emerging viruses, a constant pursuit is required to better understand these interactions.

## Author Contributions

NK, UK, and JJ conceived the concept for this review article. NK, UK, ST, and JJ wrote the manuscript. NK, UK, ST, and JJ read, edited, and reviewed the manuscript. All authors contributed to the article and approved the submitted version.

## Conflict of Interest

The authors declare that the research was conducted in the absence of any commercial or financial relationships that could be construed as a potential conflict of interest.
